# Supplementary material for: MicroPhenoDB Associates Metagenomic Data with Pathogenic Microbes, Microbial Core Genes, and Human Disease Phenotypes
Source: Genomics Proteomics Bioinformatics. 2021 Jan 6;18(6):760–72. doi: 10.1016/j.gpb.2020.11.001 (PMC8377004; doi:10.1016/j.gpb.2020.11.001)
Supplement: Supplementary Table S1 — The version or release of databases and tools used in the MicroPhenoDBconstruction [file mmc1.docx]

**Table S1 The version or release of databases and tools used in the MicroPhenoDB construction**

| **Name** | **URL** | **Year** | **Version** | **Ref.** |
| --- | --- | --- | --- | --- |
| HMDAD | http://www.cuilab.cn/hmdad | 2016 | Version 1.0 | [18] |
| Disbiome | https://disbiome.ugent.be/ | 2018 | Version 1.0 | [19] |
| IDSA guideline | https://pubmed.ncbi.nlm.nih.gov/30169655/ | 2018 | 2018 update | [22] |
| EFO | https://www.ebi.ac.uk/efo/ | 2019 | Version 3.18.0 | [30] |
| NCIT | https://www.ebi.ac.uk/ols/ontologies/ncit | 2019 | Version 19.11d | [23] |
| VFDB | http://www.mgc.ac.cn/VFs/ | 2016 | Version 1.0 | [20] |
| CARD | http://arpcard.mcmaster.ca | 2017 | Version 1.0 | [21] |
| MetaPhlAn2 | http://huttenhower.sph.harvard.edu/metaphlan2/ | 2015 | Version 2.0 | [28] |
| InterProScan | http://www.ebi.ac.uk/interpro/ | 2019 | Version 72.0 | [29] |
| NCBI-taxonomy | https://www.ncbi.nlm.nih.gov/taxonomy | 2019 | - | [26] |
| EBI tool framework | https://www.ebi.ac.uk/ | 2019 | - | [35] |
| Genome Sequence Archive | http://gsa.big.ac.cn | 2020 | - | [38] |

*Note*: CARD, Comprehensive Antibiotic Resistance Database; EBI, European Bioinformatics Institute; EFO, Experimental Factor Ontology; HMDAD, Human Microbe-Disease Association Database; IDSA, Infectious Diseases Society of America; NCBI, National Center for Biotechnology Information; NCIT, National Cancer Institute Thesaurus; VFDB, Virulence Factor Database.
