# Supplementary material for: MicroPhenoDB Associates Metagenomic Data with Pathogenic Microbes, Microbial Core Genes, and Human Disease Phenotypes
Source: Genomics Proteomics Bioinformatics. 2021 Jan 6;18(6):760–72. doi: 10.1016/j.gpb.2020.11.001 (PMC8377004; doi:10.1016/j.gpb.2020.11.001)
Supplement: Supplementary Table S2 — The analysis result by MicroPhenoDB sequence search in an existing metagenomic dataset(GSA: PRJCA000880) [file mmc2.docx]

**Table S2 The analysis result by MicroPhenoDB sequence search in an existing metagenomic dataset (GSA: PRJCA000880)**

| **Microbe species** | **Found by MicrophenoDB sequence search** | **Found in the original analysis [39]** |
| --- | --- | --- |
| *Fusobacterium nucleatum* | Yes | Yes |
| *Propionibacterium acnes* | Yes | Yes |
| *Prevotella veroralis* | Yes | Yes |
| *Aspergillus fumigatus* | Yes | Yes |
| *Haemophilus influenzae* | Yes | Yes |
| *Streptococcus cristatus* | Yes | Yes |
| *Enterococcus faecalis* | Yes | Yes |
| *Prevotella melaninogenica* | Yes | Yes |
| *Malassezia globosa* | Yes | Yes |
| *Neisseria subflava* | Yes | Yes |
| *Prevotella oulorum* | Yes | Yes |
| *Micrococcus luteus* | Yes | Yes |
| *Acinetobacter johnsonii* | Yes | Yes |
| *Streptococcus sanguinis* | Yes | Yes |
| *Veillonella parvula* | Yes | Yes |
| *Streptococcus anginosus* | Yes | Yes |
| *Serratia marcescens* | Yes | Yes |
| *Peptostreptococcus stomatis* | Yes | Yes |
| *Streptococcus salivarius* | Yes | Yes |
| *Haemophilus parainfluenzae* | Yes | Yes |
| *Neisseria flavescens* | Yes | Yes |
| *Saccharomyces cerevisiae* | Yes | Yes |
| *Prevotella pleuritidis* | Yes | Yes |
| *Corynebacterium matruchotii* | Yes | No |
| *Burkholderia cenocepacia* | Yes | No |
| *Gemella haemolysans* | Yes | No |
| *Leptotrichia wadei* | Yes | No |
| *Comamonas testosteroni* | Yes | No |
| *Lautropia mirabilis* | Yes | No |
| *Enhydrobacter aerosaccus* | Yes | No |
| *Rothia aeria* | Yes | No |
| *Ralstonia pickettii* | Yes | No |
| *Staphylococcus epidermidis* | Yes | No |
| *Stenotrophomonas maltophilia* | Yes | No |
| *Corynebacterium tuberculostearicum* | Yes | No |
| *Granulicatella elegans* | Yes | No |
| *Sphingobium xenophagum* | Yes | No |
| *Mucor racemosus* | No | Yes |
| *Mucor indicus* | No | Yes |
| *Rhizopus oryzae* | No | Yes |
| *Rhizopus microsporus* | No | Yes |
| *Aspergillus fumigatus* | No | Yes |
| *Porphyromonas* | No | Yes |

*Note*: GSA, Genome Sequence Archive.
